# Biocontrol Potential of Serratia Marcescens (B8) and *Bacillus* sp. (B13) Isolated from Urban Mangroves in Raposa, Brazil

**DOI:** 10.3390/life13102036

**Published:** 2023-10-11

**Authors:** Érima Jôyssielly Mendonça Castro Pereira, Érika Alves da Fonsêca Amorim, Felicia Maria Melo Aragão, Wallison de Souza Câmara, Maria Carvalho Araújo, Carlos Drielson da Silva Pereira, Leo Ruben Lopes Dias, Wolia Costa Gomes, Amanda Silva dos Santos Aliança, Joicy Cortez de Sá Souza, Luís Cláudio Nascimento da Silva, Rita de Cássia Mendonça de Miranda

**Affiliations:** 1 Programa de Pós-Graduação em Biologia Microbiana, Universidade Ceuma, São Luís 65075-120, Brazil; 2 Programa de Pós-Graduação em Biodiversidade e Biotecnologia da Amazônia, Universidade CEUMA, São Luís 65075-120, Brazil; erikaramalho@yahoo.com.br (É.A.d.F.A.);; 3 Programa de Pós-Graduação em Odontologia, Universidade Ceuma, São Luís 65075-120, Brazil; 4 Programa de Pós-Graduação em Meio Ambiente, Universidade Ceuma, São Luís 65075-120, Brazil; felicitaaragao@gmail.com (F.M.M.A.); souzawallison292@gmail.com (W.d.S.C.);; 5 Programa de Pós-Graduação em Gestão de Programas e Serviços de Saúde, Universidade Ceuma, São Luís 65075-120, Brazil

**Keywords:** microbial bioprospecting, biocontrol, secondary metabolite, mangrove, phytopathogen

## Abstract

This study analyzed the antifungal potential of 16 bacterial strains isolated from mangrove sediment. Bacterial selection was conducted in a solid medium. This was followed by the production and extraction of metabolites using ethyl acetate to evaluate chitinase production, antifungal activity, and toxicity toward *Allium cepa* and *Tenebrio molitor*. Bacterial strains B8, B11, and B13 produced the largest inhibition halos (>30 mm) toward *Fusarium solani*, *Fusarium oxysporum*, and *Rhizoctonia solani* fungi. Strains B1, B3, B6, B8, B11, B13, B14, and B16 produced chitinases. In assays using liquid media, B8 and B13 produced the largest inhibition halos. Exposing the fungal inocula to metabolic extracts of strains B6, B8, B11, B13, B14, B15, and B16 caused micromorphological alterations in the inocula, culminating in the inhibition of *R. solani* sporulation and spore germination. Toxicity tests using *Allium cepa* and *Tenebrio molitor* revealed that the metabolites showed low toxicity. Six of the bacterial strains were molecularly identified to species levels, and a further two to genus level. These included *Serratia marcescens* (B8), which exhibited activity in all tests. Mangroves provide a useful resource for the isolation of microorganisms for biocontrol. Among the isolates, *Serratia marcescens* and *Bacillus* spp. showed the greatest potential to produce metabolites for use as biocontrol agents in agriculture.

## 1. Introduction

Agriculture is one of the most important sectors for the economy, as it encompasses several resources of commercial interest [1,2]. The control of pests, which affect both crops and livestock, is one of the challenges affecting agricultural productivity [3]. Phytopathogenic organisms potentially hamper production as they reduce the quantity and quality of agricultural output [4,5]. Agricultural practices often rely on the intensive use of pesticides, raising significant concerns in terms of human health, environment, and sustainability. Therefore, finding safer and more sustainable alternatives has become a priority [6]. Reducing the use of pesticides and implementing biocontrol have gained prominence as fundamental strategies for addressing the challenges associated with modern agriculture. Biocontrol uses living organisms or their products to menage pests and disease development, reducing reliance on synthetic chemicals. This practice is based on a deep understanding of agricultural ecosystems and the natural balance among species [1,2,6].

The environment can be a resource for the discovery of new biologically active compounds, considering the diversity of organisms with different metabolic potentialities. This fact can lead to new research and provide the development of biologically active products [7,8]. Mangroves, with a global area of ca. 18.1 million ha [9,10,11,12], are a source of microbial prospecting because they represent a transitional state that constitutes an adaptative strategy. They produce organisms with interesting metabolic adaptations that can be exploited in the biotechnological context [13,14,15,16,17]. These sediment-dwelling organisms produce primary and secondary metabolites with essential roles in nutrient cycling and the degradation of organic matter [12,18,19,20].

The metabolites produced by microorganisms play a fundamental role in their development and interaction with the environment [21], and they represent a basis for the development of products of biotechnological interest. The biocontrol activity of *Metharizium anisoplae*, *Trichoderma harzianum*, and *Bacillus thuringiensis* via the production of bioactive substances, such as enzymes and antimicrobial compounds, has been demonstrated [12,21,22,23]. One such biocontrol mechanism is the release of metabolites that affect the organic compounds of pathogenic organisms. The fungal cell wall contains chitin as its main component, and bacterial chitinases lyse the cell walls of living and dead fungi [24,25]. Chitinases have received considerable attention owing to their potential use as biocontrol agents [26]. They have been used in biological research to generate protoplasts that can degrade fungal cell walls [26,27].

Toxicity studies are essential for determining whether metabolites can be used as biocontrol products. *Allium cepa* is used to directly evaluate the cytotoxic, genotoxic, and mutagenic activity of different compounds [28,29,30]. *Tenebrio molitor* larvae are widely used as an alternative to vertebrates, reducing costs and ensuring the reliability of results for evaluating insecticidal activity and cytotoxicity [28,29]. Compared to using other animals, using *Tenebrio molitor* larvae provides more a direct method of determining toxicity [30].

Biocontrol, a sustainable alternative to the use of pesticides for controlling pests [31], provides advantages and economic benefits related to preserving the environment and reducing the use of pesticides and their harmful effects on health and the environment [3]. Thus, these environments have become a source of prospective biocontrol organisms.

Given the need to search for microorganism-derived bioactive compounds capable of inhibiting pathogenic agents, we aimed to bioprospect microorganisms isolated from the sediment of the Mangue Seco region, a mangrove region located in the municipality of *Raposa* (Maranhão State, Brazil). Further, we evaluated the biocontrol potential of promising isolates against phytopathogenic organisms affecting the agricultural sector.

## 2. Materials and Methods

### 2.1. Microorganisms

Sixteen microbial isolates obtained from mangrove sediment at Mangue Seco beach, Raposa (MA, Brazil; 2°25’22” S, 44°06’10” W) were studied [15]. The biocontrol assay used five phytopathogenic fungi obtained from the Phytopathogen Culture Collection (State University of Maranhão, São Luís, Brazil; Table 1).

The fungal isolates were prepared via serial dilution (11). The phytopathogenic fungi were activated using liquid potato dextrose (PD) medium and maintained in Petri dishes containing potato dextrose agar medium at 4 °C.

### 2.2. Biological Activity

#### 2.2.1. Solid Media Assay

Bacterial antifungal activity was evaluated using agar diffusion on solid potato dextrose agar (PDA) [32]. Bacteria were incubated at 28 °C for 5 d in nutrient agar medium, and 6 mm plugs of each strain were transferred into a new PDA containing a growing mycelium of the fungus (fungal inoculum, 5 × 10^5^ CFU/mL). This test was performed in triplicate. After 24 h of incubation at 30 °C, the diameter (in mm) of the inhibition halo of each block was measured along the longest line dividing the block of glucose in half. The Matsuura scale [33] was used to classify the results. The arithmetic mean and standard deviation were obtained. The results are expressed as mean inhibition zone diameter (IDZ; mm).

#### 2.2.2. Submerged Fermentation

To extract the active metabolites produced by the bacteria, submerged fermentation was performed in Erlenmeyer flasks (250 mL) containing 50 mL potato dextrose broth (PDB). The results were standardized against the 0.5 MacFarland standard. The flasks were incubated in a rotary incubator at 30 °C for 5 d. The samples were then filtered through a 22 µm membrane to assess their biological activity.

##### Active Metabolite Extraction

A liquid–liquid approach was used to extract the metabolites from the fermented samples [34]. After the 5 d fermentation period, the supernatant was vacuum-filtered, and the metabolic extract was separated, centrifuged at 150 rpm for 5 min, and filtered again through a 22 μm membrane to entirely remove the bacteria cells. For extraction, 25 mL filtrate and 25 mL ethyl acetate were added to a separating funnel; this was then shaken vigorously for 10 min, and the solution was allowed to rest for 30 min. The organic phase containing the analytes was then collected. The solvent was removed via evaporation using a rotary evaporator. Product yield was quantified in mg/L. To evaluate biological activity, the extract was resuspended in dimethyl sulfoxide (DMSO) to a concentration of 1 mg/mL.

##### Liquid Medium Test—Agar Diffusion

The standard agar diffusion test of the Clinical and Laboratory Standards Institute (CLSI) [35,36] was conducted to select those microorganisms capable of secreting metabolites produced in the external environment. This test involves placing 50 μL of the extracted metabolite into wells ca. 6 mm in diameter in a Petri dish previously seeded with the phytopathogenic fungi (5 × 10^5^ CFU/mL). The dish is subsequently incubated at 28 ± 2 °C for up to 72 h). This test was performed in triplicate and the inhibition halo is then observed and measured using calipers.

##### Assessment of Micromorphology Changes

The germination rate was determined to observe micromorphological changes in the spores [37]. A total of 9 mL of the fungal suspension (1 × 10^7^ conidia/mL) was added to 9 mL of each concentration of the extract standard (1000 μg of extract per mL of standard) and sterilized distilled water (control) for 3 h. Thereafter, 50 μL of the conidial suspensions was transferred to a PDA plate. After 14 h of incubation at 28 °C, 200 conidia were counted per replicate under an optical microscope to determine the number of germinated and non-germinated conidia. To evaluate vegetative growth, 0.12 cm^2^ discs of the fungal colonies (cultivated for 8 d) were transferred to the center of the Petri dishes containing PDA (control) and PDA plus the concentrations of each extract.

After 8 d of incubation at 28 °C, colonies were measured in terms of their opposite diameters. Sporulation was determined by transferring three cylindrical blocks of 0.28 cm^2^ of each fungal colony to a test tube containing 5 mL of Tween 80 (0.01%). The conidia were then counted in a Neubauer chamber [37].

### 2.3. Chitinases Detection and Quantification

Chitinase activity was investigated as follows [38]. A suspension of each strain was inoculated into a chitin–yeast extract–salt (CYS) medium at the following proportions (g/L): chitin, 5.0; yeast extract, 0.5; potassium phosphate (K_2_HPO_4_), 2.0; magnesium sulfate (MgSO_4_·7H_2_O), 1.0; iron sulfate (FeSO_4_·7H_2_O), 0.1; and agar, 10. pH was adjusted to 7.0. The isolates were inoculated into Petri dishes containing the CYS medium and incubated at 28 ± 2 °C for 24 h to observe the formation of the translucent halo around the colony, indicating that the microorganism used chitin as a substrate and degraded it.

#### 2.3.1. Chitin Production

Colloidal chitin was prepared as previously described [38]. Spore suspensions of each strain were cultured for 72 h and inoculated into CYS medium (g/L): chitin, 5.0; yeast extract, 0.5; K_2_HPO_4_, 2.0; MgSO_4_·7H_2_O, 1.0; and FeSO_4_·7H_2_O, 0.1. pH was adjusted to 7. After 36 h, the culture filtrate was collected and used as an enzyme source.

#### 2.3.2. Chitinase Assay

Extracellular chitinase activity was determined by incubating 1 mL of the crude enzyme with 1 mL of 1% chitin in a 0.05 M phosphate buffer, pH 7.0, at 35 °C for 1 h. After centrifuging the reaction mixture at 15 rpm and 28 °C, the quantity of N-acetyl- D-glucosamine released in the supernatant was determined by the method of Reissig et al. [30], using N-acetyl-D-glucosamine (NAG) as a standard. The quantity of NAG present in a 0.5 mL aliquot of the supernatant was determined by adding 0.1 mL K_2_B_4_O_7_, followed by boiling for 3 min in a water bath. The tubes were cooled, and 3 mL of *p*-dimethyl-amino benzaldehyde (C_9_H_11_NO) was added. Absorbance was read within 10 min at 585 nm against a blank prepared using distilled water without the enzyme. One unit (U) of chitinase is defined as the amount of enzyme that releases 1 μM N-acetyl-D-glucosamine per hour under the conditions used here.

*Fusarium oxysporum* was used as the source of chitin. For this purpose, *F. oxysporum* was cultivated in Czapek-Dox broth for 15 d. The fungal mat was then harvested and autoclaved at 12 °C and 15 lbs for 20 min. The autoclaved fungal mat was washed twice with sterile distilled water and dried in an oven at 80 °C to a constant weight [12]. The dried fungal mat was pulverized and used as a chitin source (2 g/L) to produce chitinase. The strains’ utilization of fungal tapeworms for chitinase production was determined using three sets of flasks: (A) CYS medium in which colloidal chitin was replaced with fungal tapeworms (2 g/L); (B) CYS medium supplemented with tapeworm fungus (2 g/L); and (C) control with CYS medium without a fungal mat.

### 2.4. Molecular Identification

Molecular identification of the promising bacteria isolates was performed using the 16s ribosomal technique [12]. The primers used for PCR amplification of the 16S rRNA gene (526 bp amplicon) were BSF-8 (5′-AGAGTTTGATCCTGGCTCAG-3′) and BSR-534 (5′-ATTACCGCGGCTGCTGGC-3′). The Quantitect SYBR Green kit (Qiagen, Hilden, Germany) was used for real-time PCR (50 mL total volume) with PCR buffer and 0.2 mM of each primer. Samples (1 and 5 mL) were tested using real-time PCR. The amplification profile was 95 °C for 15 min, followed by 40 cycles at 94 °C for 30 s, 55 °C for 30 s, and 72 °C for 30 s. For this, the newly grown samples were incubated for 2 h in 2 mL of phosphate buffer saline (PBS) at 27 °C and then centrifuged at 7500 rpm for 10 min. The supernatant was discarded after centrifugation. The pellet was resuspended in 180 µL of lysosine (200 µg/mL) and incubated for 30 min at 37 °C. Then, 20 µL of proteinase K and 200 µL of Buffer AL (provided by the manufacturer) were added, followed by homogenization and incubation for 30 min at 56 °C. This was followed by another 15 min of incubation at 95 °C. After centrifugation, 200 µL of absolute ethanol was added, followed again by homogenization and centrifugation. The samples were transferred to a purification kit (QIAmp DNA Mini Kit) and centrifuged for 1 min at 8000 rpm. Subsequently, the filtrate was discarded, and 500 µL of AW2 buffer solution (QIAmp) was added, followed by centrifugation for 3 min at 1400 rpm. The filtrate was discarded again, followed by centrifugation for 1 min at 1400 rpm; 50 µL of Buffer AE (provided by the manufacturer) was added, and the product was incubated for 1 min and centrifuged again for 1 min at 8000 rpm. The final step was repeated once. After this process, the DNA was quantified by optical density in a spectrophotometer (NanoDrop ND-1000 UV-Vis) and frozen at −20 °C.

The samples were added to the wells of an agarose gel immersed in a Tris–borate–EDTA buffer solution, and electrophoresis was performed for 40 min. The gel was then stained with a nucleic acid staining solution (0.1 µL/mL) from INI Safe Dye (20,000×) for 40 min, protected from light for observation in an ultraviolet light transilluminator.

The PCR products were sequenced and analyzed using Mega and BLAST (NCBI) [39]. The 16S rRNA gene sequence obtained by PCR was analyzed and manually aligned with strains of the genus found in the GenBank/NCBI database, using Clustal-X. The nucleotide sequence was submitted to GenBank and deposited under accession number MT634691.

### 2.5. Assessment of Toxicity in a Plant Model (Allium cepa)

Sterilized and fungus-free seeds of *Allium cepa* (n = 50 seeds per plate) were distributed in Petri dishes (50 × 20 mm) lined with filter paper (90 mm). The sets of seeds were sprayed with the metabolites of bacterial isolates B8 or B13, or with DMSO (0.05%) [34]. Subsequently, the plates were incubated at room temperature in light to observe germination. Slides were prepared from the roots collected when they reached 2 cm in length. Toxicity was analyzed based on germination and variation in average root length (VCMR) [40].

The cells were observed under an optical microscope (40×), and 4000 cells per treatment were analyzed to evaluate all mitotic stages (interphase, prophase, metaphase, anaphase, and telophase). The mitotic index (MI) was calculated using the following equation [41]:

MI = m/T × 100(1)

where m is the number of cells in mitosis and T is the total number of cells.

### 2.6. Assessment of Toxicity in an Alternative Model

To assess whether the metabolites presented toxicity in insects, the larva of *Tenebrio molitor* was used. Based on a test–control ratio, 10 larvae of the *Tenebrio molitor* were used to test each metabolic extract obtained via liquid extraction. Petri dishes were previously labeled with sample information, one for each sample and control group, provided by phosphate saline buffer injected with PBS (phosphate saline), which contains a saline solution compatible with that of the human body.

The larvae were purchased from Biofábrica S. A. (São Luís, Brazil). The larvae were separated, sanitized, and standardized to the same size, weight, and stage. Then, 10 larvae were placed on each plate on ice so that they remained in hibernation stage, which facilitated handling. Each larva received 10 μL of a cell-free metabolic fluid sample injected into its third ring. Subsequently, the larvae were stored at room temperature and observed every 24 h for 10 d.

### 2.7. Statistical Analysis

Results are expressed as the mean and standard deviation and were analyzed using GraphPad Prism 5.0. Statistical evaluation was performed using analysis of variance (ANOVA). The data obtained for the toxicity assay using the plant model were first subjected to Kolmogorov–Smirnov testing. The Kruskal–Wallis test was used to compare the treatments with the positive control. The survival curve was plotted based on Kaplan–Meier analysis, and the results were analyzed using the log-rank test. In all tests, a significance level of 95% was considered to indicate significant difference (*p* < 0.05).

## 3. Results

### 3.1. Biological Activity

#### Solid Media Assay

The test in the solid medium was performed against phytopathogenic fungi, and the results after measuring the halos are shown in Table 2. We observed the formation of inhibitory halos by eight active bacteria against crop pathogens.

Of the 16 bacterial strains isolated, 8 were active against phytopathogenic fungi. Among these, isolates B06, B8, B11, B13, and B14 were the most promising, with halos > 30 mm. Among the four isolates mentioned, bacterium B06 exhibited the highest activity against *S. rolfsii* (37.6 mm ± 0.5 mm) and *R. solani* (34 mm ± 1 mm). Additionally, B8 exhibited an inhibition halo of 35 mm ± mm 1 against *R. solani*, while B11 was active against *F. oxysporum*, with a halo of 34.6 mm ± 0.5 mm. Finally, isolate B13 was active against *F. oxysporum*, with an inhibition halo of 36.2 mm ± 2.8 mm, and against *F. solani*, with a halo of 33.3 mm ± 2.8 mm.

### 3.2. Submerged Fermentation

#### 3.2.1. Secondary Metabolite Extraction

**Figure 1:**
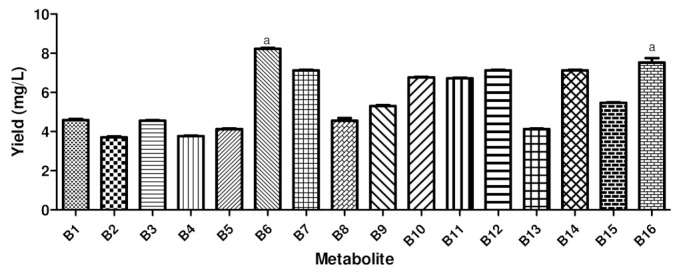
The final yield of metabolites extracted by ethyl acetate, after production in submerged fermentation, of the 16 bacteria isolated from mangrove sediment. a: statistically significant difference at *p* < 0.05.

The fractions of the final yield of the extracted bioactive substances (in mg) were analyzed to quantify the metabolites extracted from the liquid medium via liquid–liquid separation. We concluded that obtaining a desirable quantity of metabolites using this simple and cheap technique is feasible, since the promising samples produced final yields of 8.2 mg/L (B6), 4.3 mg/L (B8), 5.6 mg/L (B11), 4.1 mg/L (B13), 7.1 mg/L (B14), 5.5 mg/L (B15), and 7.7 mg/L (B16) (Figure 1). When the yields obtained from different microorganisms were compared, a statistically significant difference was observed in the metabolites produced by bacteria B6 and B16.

#### 3.2.2. Liquid Medium Test—Agar Diffusion

The agar diffusion assay was performed with the bacterial strain that exhibited activity in the earlier test in liquid medium to evaluate its ability to secrete secondary metabolites into the medium. The activity obtained at this stage can be assessed from the formation of inhibition halos in the presence of agricultural pathogens (Table 3).

Bacteria (B6, B8, B13, B11, B14, B15, and B16) secreted secondary metabolites into the external environment, demonstrating activity against phytopathogenic fungi tested in the liquid medium test and the agar diffusion Assay. In this assay, the isolate B13 stands out, as it was capable of secreting the metabolites that inhibited all of the phytopathogenic fungi tested, with halos of 31 mm ± 6.9 mm against *F. solani*; 29.3 mm ± 1.1 mm against *F. oxysporum*; 24.6 mm ± 0.5 mm against *M. phaseolina*; 19.3 mm ± 1.1 against *R. solani*; and 23.3 mm ± 1.5 mm against *S. rolfsii*.

#### 3.2.3. Assessment of Micromorphological Change

**Figure 2:**
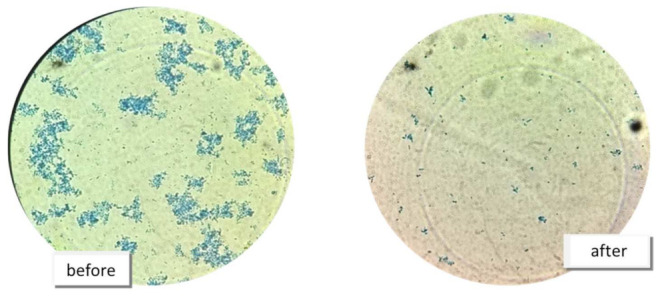
Observed number of spores of the fungus *R. solani* before and after exposure to the metabolite produced by the B8 bacteria isolated from mangrove sediments.

All of the microbial extracts inhibited the germination of *R. solani* (Table 4). The spore counts of the phytopathogenic inoculum after immersion in the metabolic extracts were lower than those of the controls, and they produced significantly fewer germinated spores (Figure 2). This leads us to infer that the metabolites are promising fungicidal agents and can be used as alternative sources to pesticides.

#### 3.2.4. Chitinase Investigation

**Figure 3:**
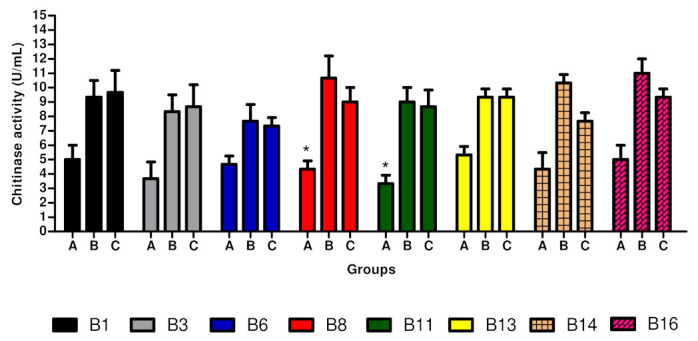
Production of chitinase by mangrove bacteria in different substrates. A, CYS medium in which colloidal chitin was replaced by the dried fungal mat of *Fusarium oxysporum*. B, CYS medium supplemented with a dried fungal mat of *Fusarium oxysporum*. C, Control only with CYS medium without any fungal mat. * *p* < 0.01.

The use of the dried *F. oxysporum* for chitinase production was studied (Figure 3). Sixteen bacterial strains were tested for chitinase production. Isolates with the potential to degrade chitin were selected. Of these, eight bacterial isolates (B1, B3, B6, B8, B11, B13, B14, and B16) produced chitinases, as evidenced by the formation of a halo in the culture medium. These eight strains produced chitinase under the three tested conditions; in particular, B8 exhibited chitinase activity of ±11 U/mL. Enzyme production decreased when the strain was grown in the CYS medium, in which colloidal chitin was replaced by dry fungal mats as a source of chitin for all bacteria tested. A slight increase in chitinase production was observed in the CYS medium amended with dried fungal mats.

#### 3.2.5. Molecular Identification

For those isolates that showed promise in biological assays, molecular identification was performed using the 16s ribosomal technique. This revealed similarity >97% for each compatible strain (Table 5). Subsequently, the isolate was identified macroscopically. Evolutionary analyses were performed using MEGA X. Only those isolates exhibiting promising activity against phytopathogenic fungi were identified in this way. We thereby identified 6 (B1, B3, B6, B8, B11 E B16) of the 16 strains to species level and a further 2 (B13 E B14) to genus level (Table 5).

#### 3.2.6. Assessment of Toxicity in a Plant Model (*Allium cepa*)

After the germination of *A. cepa* seeds (7 d) sprayed with the metabolites of B8 and B13, or with the positive control (0.05% DMSO), the secondary metabolites of *S. marcescens* (B8) and *Bacillus* sp. (B13) exerted few effects throughout the phases of cell division (mitosis), indicating low toxicity (Table 6).

#### 3.2.7. Assessment of Toxicity in an Alternative Model

**Figure 4:**
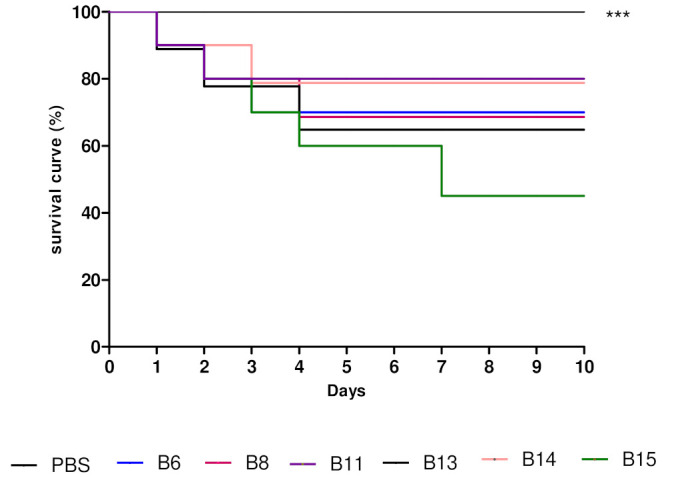
Assessment of the toxicity of metabolites produced by bacterial strains isolated from mangrove sediment, using *Tenebrio molitor* larvae. *** *p* < 0.01.

Using the alternative model (*T. molitor*) to evaluate toxicity produced a survival curve for the larvae over 10 d (Figure 4); this curve suggests that the secondary metabolites produced by *B. humi* (B11) exhibit good efficacy and low acute toxicity. The metabolites produced by *M. yunnanensis* (B06) and *S. marcescens* (B8) exhibited medium to moderate toxicity (Figure 4).

Spearman’s correlation analysis of the metabolite toxicity data in relation to the PBS control revealed a statistically significant difference between all of the tested metabolites. This test enabled us to predict cell viability and the need for future toxicity tests in cells or in more complex models.

## 4. Discussion

We evaluated the antifungal potential of 16 bacterial species isolated from mangrove sediments in Raposa, Maranhão, Brazil. Owing to its transitional dynamics, this ecosystem shows promise for prospecting microorganisms of biotechnological interest [16]. The results obtained in this study corroborate prior findings [42], in which different microbial groups (both Gram-positive and Gram-negative bacteria) were isolated from mangrove sediments in an estuarine region of India.

These microorganisms produce primary and secondary compounds of biotechnological interest that can be used in agriculture and in the food and pharmaceutical industries. Here, the isolated bacteria showed promising production of the metabolites of interest in the PDA medium, and the extraction method using ethyl acetate was effective. According to Meyer et al. [9], this solvent is advantageous for extracting metabolites because of its medium polarity, high efficiency, and low cost compared to other solvents, such as isoamyl alcohol, hexane, or methane. The bacterial extracts evaluated here showed considerable yield, comparable to the results of Wang et al. [43], who characterized the effects of secondary metabolites of *Streptomyces* sp. against phytopathogens using the liquid–liquid technique, with ethyl acetate as a solvent.

The biological activity assays used here revealed that the isolated bacteria are promising for the biocontrol of phytopathogenic fungi. An assay using a solid medium was used for the initial screening; this revealed eight bacterial strains as promising candidates for further study. These results were validated by the assay using liquid medium containing the extracted metabolites. Amorim et al. [44] used a solid medium assay for microbial screening to select microorganisms capable of producing and secreting metabolites of interest, describing the formation of inhibition halos using both solid medium (agar block) and the agar diffusion test (liquid medium).

Our findings from the chitinase assay corroborate the results of the study by Silva et al. [45], who discovered that six chitinase-producing endophytic bacteria induce antagonism against phytopathogenic fungi and insects. The same bacterial species isolated in this study were used in that study: *S. marcescens*, *P. aeruginosa*, and *B. cereus*. Here, *S. marcescens* stands out, corroborating the work of Pommer et al. [41], a study on the enzymatic hydrolysis of chitin, in which it was among the most promising bacteria in producing chitinase. Those authors found that a combination of enzymes from a filamentous fungus and *S. marcescens* could completely degrade high concentrations of chitin. *Bacillus* has been described as one of the main producers of several extracellular lytic enzymes, including cellulases, amylases, proteinases, and chitinases, which can be used for multiple biotechnological applications [45].

We observed the inhibition of fungal spore germination by the metabolites extracted from the isolated bacteria. Of the eight bacterial species tested, seven showed efficacy in inhibiting the germination of phytopathogenic fungal spores and may be considered promising biocontrol agents. Knowledge of the action of extracts on the germination of fungal isolates is essential when used in biocontrol, as germination is the first step in fungal infection, which begins with the adhesion of spores to the surface of plant parts [5,30,42]. Da Silva et al. [46] reported the importance of researching metabolites of bacteria isolated from soil for biological control. The authors showed the control of rubber tree disease by actinobacteria metabolites. Khadiri et al. [47] showed the antagonistic effect of *Bacillus cereus* against several phytopathogenic fungi of citrus fruits. The authors used the same methodology used in this work to assess the ability of the bacteria to inhibit the germination of fungal spores.

Our findings reveal, in particular, that bacterial isolates B06, B8, and B11 inhibited the germination of spores of *R. solani*. This is of paramount importance because this is the phytopathogen that most arouses concern and interest because of its occurrence in various important crops, and because of the increase in its resistance owing to the indiscriminate use of pesticides [46]. Therefore, the present work is relevant as it offers a glimpse of metabolites from mangrove isolates capable of inhibiting *R. solani* (FP4). Some authors report microbial metabolite activity against *R.solani*. Arasu and Al-Dhabi [48] reported the antagonistic action of an extract of the fungus *Paecilomyces formosus* against several phytopathogenic fungi, including two species of Rhozictonia. The authors evaluated in vitro and in vivo activity and obtained a concentration of 10^8^ fungal spores as the most effective for inhibiting the growth of *R. solani* in tomato. Silva et al. [45] reported the antagonistic activity of three Bacillus species against *R. solani* that causes web in cowpea (*Vigna unguiculata* (L.) Walp.)

Toxicity studies are important because they determine whether the metabolites can be used as biocontrol products. *A. cepa* is used to evaluate the cytotoxic, genotoxic, and mutagenic activity of different compounds [28,29] as it offers a more direct method to verify whether the compound is toxic [30]. Here, the low toxicity of the promising compounds was verified in that they did not stimulate cell division with chromosomal alterations, suggesting that the products from these microorganisms can be used. Other authors have evaluated secondary metabolite toxicity using an *A. cepa* model. Eckert et al. [49] evaluated the genotoxicity and mutagenicity of plant extracts using *A. cepa* as a model. Madic et al. [50] evaluated the genotoxicity of the five plana extract with activity against entomopathogenic insects.

*Tenebrio molitor*, known as the yellow mealworm, is a polyphagous insect that infests cereals, flour, bran, and pasta worldwide. Both larvae and adults of the pest cause significant damage to stored products [45]. *T. molitor* larvae is widely used as an alternative to vertebrates to reduce costs and ensure reliability. It is widely used to evaluate antimicrobial and insecticidal activity and cytotoxicity [28,29] as it provides a direct way to evaluate toxicity [30,42]. Eski et al. [51] and Walkowiak-Nowicka et al. [52] used *T. molitor* larvae to evaluate the potential of four plant metabolites with antifungal activity. The authors used the larval stage of the insect and observed several morphological damages in it.

## 5. Conclusions

Based on these findings, the mangrove ecosystem in this region is endowed with cultivable microorganisms with biotechnological potential and antibiotic activity against agricultural pathogens. This work reveals that it is possible to extract the metabolites secreted by these promising isolates using low-cost and efficient methods.

## Figures and Tables

**Table 1:** Isolate code, scientific name, and phytopathology of the phytopathogenic fungi used to evaluate the biological activity of the related metabolites.

Isolate Code	Pathogen Name	Reported Disease
FP1	*Fusarium solani*	dry root rot
FP2	*Fusarium oxysporum*	fusarium wilt
FP3	*Macrophomina phaseolina*	charcoal rot
FP4	*Rhizoctonia solani*	root rot
FP5	*Sclerotium rolfsii*	sclerotium wilt

**Table 2:** Antimicrobial activity against phytopathogenic fungi of bacterial strains isolated from mangrove sediment (inhibition zone in mm).

Isolate Code	*F. solani*	*F. oxysporum*	*M. phaseolina*	*R. solani*	*S. rolfsii*
B1	0	0	0	0	0
B2	0	0	0	0	0
B3	0	0	0	0	0
B4	0	0	0	0	0
B5	0	0	0	0	0
B6	19.3 ± 1.1	24 ± 1.7	30.6 ± 4.9 **	34 ± 1.0	37.6 ± 0.5 **
B7	0	0	0	0	0
B8	22.6 ± 2.0	32.6 ± 1.1 **	32.6 ± 2.0 **	35 ± 1.0 **	24 ± 1.0
B9	0	0	0	0	0
B10	0	0	0	0	0
B11	11.6 ± 2.8	34.6 ± 0.5 **	24.3 ± 1.1	30.3 ± 0.5	13.3 ± 2.8
B13	33.3 ± 2.8 **	36.6 ± 2.8	28.6 ± 1.1	32.3 ± 2.5 **	26 ± 1.7
B14	29 ± 1.0	22 ± 1.7	13.3 ± 2.8	18.3 ± 2.8	20.3 ± 0.5
B15	21 ± 1.0	17.3 ± 2.5	13.3 ± 2.8	20.3 ± 0.5	15.3 ± 0.5
B16	20.3 ± 0.5	17.3 ± 3.2	0	16.3 ± 1.1	0
B17	0	21 ± 1.0	17.6 ± 2.5	0	29.3 ± 1.1

** *p* < 0.01.

**Table 3:** Diameters of zones (in mm) of inhibition (mean and standard deviation), expressing the antimicrobial activity of metabolites produced and extracted from bacteria isolated from mangrove sediment against phytopathogenic fungi.

Isolate Code	*F. solani*	*F. oxysporum*	*M. phaseolina*	*R. solani*	*S. rolfsii*
B06	0	21.3 ± 1.1	23.3 ± 2.8	27.6 ± 2.5	0
B8	0	22.3 ± 2.5	25.3 ± 0.5	27.6 ± 0.5	0
B11	0	21.3 ± 1.1	0	0	0
B13	31 ± 6.9 **	29.3 ± 1.1 **	24.6 ± 0.5	19.3 ± 1.1	23.3 ± 1.5
B14	26 ± 1.0	20.3 ± 0.5	0	17.3 ± 2.5	0
B15	0	0	0	13 ± 1.7	0
B16	0	0	0	13.6 ± 1.5	0

** *p* < 0.01.

**Table 4:** Number of spores germinated in the control and after contact with the metabolite produced and extracted from the isolated bacteria.

Isolate Code	*F. solani*	*F. oxysporum*	*M. phaseolina*	*R. solani*	*S. rolfsii*
Control	18 **	15 **	10 **	12 **	8 *
B6	0	0	1	0	0
B8	0	1	0	0	0
B11	0	2	0	0	0
B13	1	1	1	2	0
B14	3	2	0	2	0
B15	0	0	0	3	0
B16	0	0	0	0	0

** *p* < 0.01; * *p* > 0.05.

**Table 5:** Codes of isolates, Gram classification, degree of similarity, molecular identification, and NCBI code of promising bacteria isolated from mangrove sediment.

Isolate Code	Gram	Similarity	Identification	NCBI Code
B1	G^−^	99.20%	*Achromobacter xylosoxidans*	D1454195
B3	G^−^	95.84%	*Pseudomonas* sp.	1ABAAA38
B6	G^+^	97.83%	*Micrococcus yunnanensis*	6A934C15
B8	G^−^	99.3%	*Serratia marcescens*	92C94ED7
B11	G^+^	99.5%	*Bacillus humi*	547CB45C
B13	G^+^	97%	*Bacillus* sp.	n/c
B14	G^+^	97%	*Bacillus* sp.	n/c
B16	G^+^	99.5%	*Bacillus cereus*	F292A36B

**Table 6:** Mitotic phases and mitotic index (MI) (mean ± standard deviation) in A. cepa radicle cells subjected to 7 days incubation with a metabolite produced by bacteria isolated from mangrove sediment. The results were calculated with mean and standard deviation and a statistical test was applied, adopting statistical difference (*p* < 0.05).

Mitotic Phases	Positive Control	B8	B13
Interface	1184.75 ± 636.17	456.75 ± 307.85	963.5 ± 581.45
Prophase	8.25 ± 7.13	6.75 ± 5.46	7.25 ± 2.06
Anaphase	0.0 ± 0.0	0.25 ± 0.05	0.75 ± 0.5
Metaphase	0.0 ± 0.0	2 ± 1.41	2.75 ± 3.2
Telophase	0.0 ± 0.0	0.25 ± 0.05	5.5 ± 2.8
MI	27.1%	2.85%	2.6%

## Data Availability

Ribosomal 16S sequence data was deposited at https://www.ncbi.nlm.nih.gov/guide/sequence-analysis/.
